# Thoracoscopic surgery approach to mediastinal mature teratomas: a single-center experience

**DOI:** 10.1186/s13019-020-1076-7

**Published:** 2020-02-12

**Authors:** Lu Huu Pham, Diep Ke Trinh, Anh Viet Nguyen, Lanh Sy Nguyen, Dung Thanh Le, Dinh-Hoa Nguyen, Hung Quoc Doan, Uoc Huu Nguyen

**Affiliations:** 1 Cardiovascular and Thoracic Center, Viet Duc University Hospital, Hanoi, 100000 Vietnam; 2 Hanoi Medical University, Hanoi, 100000 Vietnam; 3 Department of Anesthesia, Viet Duc University Hospital, Hanoi, 100000 Vietnam; 4 Department of Pathology, Viet Duc University Hospital, Hanoi, 100000 Vietnam; 5 Department of Radiology, Viet Duc University Hospital, Hanoi, 100000 Vietnam; 6 Department of Trauma and Orthopaedic Surgery, Viet Duc University Hospital, Hanoi, 100000 Vietnam

**Keywords:** Mediastinal mature teratomas, Thoracoscopic surgery

## Abstract

**Background:**

Mediastinal mature teratomas are rare tumors with diverse surgical approaches. The aim of this study is to review our experience of thoracoscopic surgery management in patients with teratomas.

**Methods:**

We retrospectively reviewed 28 consecutive patients with mediastinal mature teratomas who underwent thoracoscopic surgery at Viet Duc University Hospital from January 2008 to August2018. Patients were divided into 2 groups with 2 types of thoracoscopic surgery, closed thoracoscopic surgery (CTS) group and video-assisted thoracoscopic surgery (VATS) group. The selection of sugical approach was based on sizes, locations and characteristics of tumors. Post-operative outcomes were assessed and compared between these 2 groups.

**Results:**

There were 14 female and 14 male patients with a median age of 41.2 ± 13.8 years. A total of 22 teratomas were located on the right side of the chest cavity and 6 on the left side. We performed CTS in 21 patients (75%) and VATS in 7 patients (25%) for tumor resection. There were 3 cases (10.7%) required conversion to minithoracotomy (5 cm in incision length). Skin appendages accounted for the highest rate (96.4%) in pathology. There was no record of mortality or tumor recurrence detected by computerized tomography.

**Conclusion:**

A thoracoscopic surgery for a mediastinal mature teratoma was a feasible choice. Challenging factors such as large tumors, intraoperative bleeding and strong tumor cell adhesion were considered handling by conversion to mini-thoracotomy that could ensure safety procedures and complete removal of tumors. Extraction of tumor contents might be performed for patients with large mature cystic teratomas to facilitate thoracoscopic surgery.

## Introduction

Mediastinal mature teratomas (MMT), accounting for 5 to 10% of all mediastinal tumors [1], are usually located in the anterior mediastinum,. They can be cured with complete surgical resection with favorable prognosis [2]. Open thoracotomy is still preferred in cases with large or malignant tumors [3]. Sato et al. compared the efficacy and safety of mediastinal tumor resection in children used open thoracotomy (OT) versus VATS and suggested that VATS was the less invasive method because of its fewer blood transfusion requirements and shorter thoracic drainage duration and hospital stay [4]. VATS was widely used for the resection of mediastinal masses [5, 6], with many successful VATS procedures to remove benign mediastinal tumors have been reported [6]. With the development of science and technology in operative techniques, thoracoscopic surgery such as closed thoracoscopic surgery (CTS) or video-assisted thoracoscopic surgery (VATS) has become more popular [7, 8] and currently has been accepted and widely deployed by many thoracic surgeons in the world. However, the roles of tumor sizes, grades of adhesion and surgical experience in thoracoscopy decision were remained unknown [9, 10]. The aim of this study was to clarify these aspects in thoracoscopic surgery for mediastinal mature teratomas that has been applied at the Department of Cardiovascular & Thoracic Surgery – Viet Duc University Hospital.

## Materials and methods

We retrospectively reviewed 28 consecutive patients with mediastinal mature teratomas who underwent thoracoscopic surgery at Viet Duc University Hospital from January 2008 to August 2018.

### Inclusion criteria and exclusion criteria

**Fig. 1:**
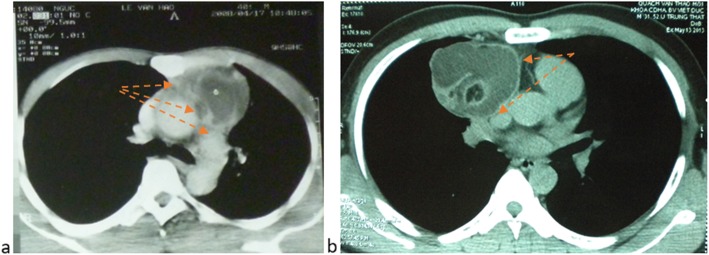
Mediastinal mature teratomas on CT-scanner. **a** Adhesion lesion with surrounding organs [arrow] – VATS conversion mini-thoracotomy and **b** clear boundary with surrounding organs [arrow]

All cases of teratomas would undergo thoracoscopic surgery if [1] diagnosis of a mediastinal mature teratoma was made on computerized tomography with these following characteristics: tumor boundary is quite clear, it is benign (based on low contrast concentration, calcification within the tumor on CT) or include mixed components that is consistent with a mature teratoma; and [2] tumor diameter is less than 10 cm. Patients do not have any contraindications for thoracoscopic surgery. Patients were divided into 2 study groups with 2 types of thoracoscopic surgery: CTS group and VATS group based on sizes, locations and characteristics of tumors. VATS was used when a teratoma had at least one of these characteristics: larger than 6 cm in diameter (but still smaller than 10 cm), suspected adhesion (CT-scanner image showed that boundaries of tumor were unclear with surrounding organs) or the tumor was on the left side of the chest cavity (Fig. 1a).

### Operation technique

All patients were under general anesthesia with double-lumen intubation and CO2 insufflation of 5–10 mmHg if CTS was performed [11, 12]. The patient lied on the semilateral decubitus position. Thoracoscopic surgery was performed with CTS (skin incision < 1 cm – only used with trocars 5 mm, 10 mm or 12 mm) or VATS approach. In CTS, the surgeon stood behind the patient and used Karl-Storz endoscopic instruments to set up trocars as type IV of Sasaki [13]. The first trocar for the camera was located in 6th or 7th ICS on the posterior axillary line. Other trocars (the 2nd or 3rd trocar) were placed depending on the location of tumor that usually aligned according to the principle of “baseball diamond concept” [14] (The second one is into the 3th or 4th ICS on the same line as the first trocar. The target trocar is usually on the 3th mid-clavicular line or the 7th ICS on the anterior axillary line). In VATS, we kept placing the first trocar in the 6th or 7th ICS on the posterior axillary line as in CTS, but the 2 other trocars in CTS were replaced in VATS by a small incision which was about 2 to 4 cm in length (without spreading the ribs) in 5th ICS between linea axillaris anterior and linea axillaris posterior. This incision site was aimed to avoid scarring in anterior area and to hide the surgical scar by arm movements or bras (if patients were female), but still guaranteed it was the appropriate site to approach teratomas. We used VATS if the tumor was large (> 6 cm in diameter), had thick pleural adhesions or invasively encroached into the surrounding organs that make it difficult to be completely dissected by thoracoscopic surgery. Dissection and complete removal of tumors were performed with an electrocautery, harmonic scalpel, ligasure or silver clip (for hemostasis). In some cases, if a teratoma mainly contained fluid, we would extract its contents before dissecting and removing them. If the tumor was small, it was taken out of the chest by putting into a plastic bag, through a trocar hole or through a 2 - 4 cm skin incision extended from a trocar hole. Otherwise, alarge tumor would be divided into smaller piecies and removed through a small hole on the chest wall. The specimens were sent to pathological department. Hemostasis was managed before we finishish the operation. Usually, a pleural drainage of 32F or 18F silicone was used under the camera’s guidance to expand the lungs before removal of the trocars and closure of the surgical wounds.

### Post-operative follow-up

The following outcomes were assessed. Postoperative complications were documented. Postoperative follow-up such as drainage, X-ray examination, and diagnostic results was evaluated.

### Statistical analysis

Statistical analyses were performed using SPSS version 20.0 package (IBM Corporation, Armonk, NY, USA). Descriptive analyses were presented as median (range) or mean with standard deviation. The preoperative, intraoperative and postoperative variables between the CTS group and VATS group was compared with a χ2 (chi-square) test for categorical variables and a Student *t-*test for continuous data.

## Results

### Clinical characteristics

There were 14 female and 14 male patients (ratio = 1:1) with a median age of 41.2 ± 13.8 years (range 12–63 years). Among 28 patients with mature teratomas, 7 cases (25%) were asymptomatic at the time of surgery, and the lesions were discovered incidentally on chest X-rays and CT-scans. The most common symptoms in the mature teratoma group included chest pain (81%), chest tightness or discomfort (52.4%), cough (19.1%), and fever (9.5%).

There were 22 teratomas located on the right side of the chest cavity and 6 ones on the left side. Alpha-fetoprotein and beta-human chorionic gonadotropin levels in all 28 patients were within normal limits. A total of 8 patients underwent preoperative CT-guided aspiration. No definite cytological or pathological diagnoses were made.

### Surgical results

A total of 28 patients have undergone thoracoscopic surgery for mediastinal mature teratomas. There were 21 patients (75%) performed CTS approach, whereas 7 patients (25%) were undergone VATS for tumor resection.

All tumors were removed totally. There were 3 cases (10.7%) required conversion to minithoracotomy (5 cm) due to in-operative bleeding and adhesion of tumors. Chest tube stay is 2.5 ± 0.5 days and postoperative hospital stay is 3.5 ± 0.6 days (Table 1). We compared 2 groups with significant statistical significance for chest tube stay (*p* = 0.011) and postoperative hospital stay (*p* = 0.024).

**Table 1:** Comparison between operative methods in patients with mediastinal mature teratomas

	Total (*n* = 28)	CTS (*n* = 21)	VATS (*n* = 7)	*p*-value (CTS vs VATS)
Age (yr)	41.2 ± 13.8	40.9 ± 14.7	42.1 ± 11.7	0.836
Gender (%)				
Male	50	57.1	28.6	0.190
Female	50	42.9	71.4	
Symptom (%)				0.801
Yes	75	76.2	71.4	
No	25	23.8	28.6	
Tumor size (cm)				
Size 1	44.1	44.9	53.2	0.212
Size 2	53.2	52.1	59.7	0.265
Adhesions (%)				< 0.001
Yes	28.6	4.80	85.7	
No	71.4	95.2	14.3	
Operative time (min)	134.6 ± 24.5	131.6 ± 22.8	143.6 ± 29.1	0.270
Chest tube stay (d)	2.5 ± 0.5	2.3 ± 0.5	2.9 ± 0.4	0.011
Postoperative hospital stays (d)	3.5 ± 0.6	3.4 ± 0.6	3.9 ± 0.4	0.024
Conversion to mini-thoracotomy (%)	10.7	4.8	28.6	0.078

In our study, no serious complications occurred after operation related to surgery. The postoperative follow-up time was 70.4 ± 38.1 months (range 1–150 months). There was no record of mortality or tumor recurrence detected on computerized tomography.

### Pathological characteristics

The most common components of mediastinal mature teratomas were skin or skin appendages and nerve tissue in the ectoderm; adipose tissue, cartilage and bone in the mesoderm; and respiratory epithelium, uroepithelium and gastrointestinal epithelium in the endoderm (Table 2). The results of our study showed that the figure of tumors containing components derived from 2 of 3 sources (endoderm, mesoderm, and ectoderm) accounted for 85.7% of all tumors. Skin and skin appendages accounted for the highest rate (96.4%) of all components.

**Table 2:** Histopathology in mature teratomas

Histopathology in mediastinal mature teratoma	No of case
Ectoderm	
Skin or skin appendages	27
Nerve tissue	07
Sweat gland	06
Mesoderm	
Cartilage	02
Adipose tissue	17
Bone	04
Muscle	02
Endoderm	
Respiratory epithelium	08
Pancreatic tissue	03
Gastrointestinal epithelium	10
Uroepithelium	11
Fallopian tube	04
Thyroid	03

## Discussion

### Clinical manifestations of mediastinal mature teratomas

Mediastinal tumors are conditions without specific clinical manifestations [1, 7, 15]. The most common symptom was chest pain that accounted for 81% of all manifestations in our study. In addition, some non-specific manifestations such as chest tightness or discomfort (52.4%), cough (19.1%), and fever (9.5%) were also consistent with other studies [8, 10, 16, 17]. Although symptoms did not play the most important role in diagnosing teratomas, they might be related to contraindications for thoracoscopic resection of teratomas because of their association with surgical complications [16, 17]. Regular screening is important for detecting a tumor when its size is small, and it can be indicated with a thoracoscopic surgery that will benefit the patient. In our study, there were no differences between CTS and VAST in relationship with clinical symptoms.

### Indication of thoracoscopic surgery for teratomas

**Fig. 2:**
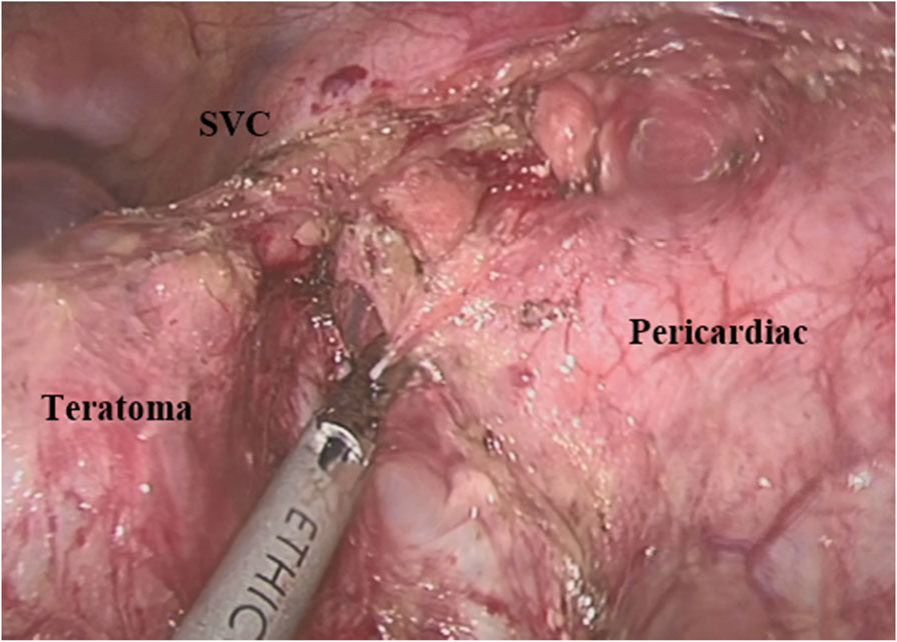
In-operative lesion with dissection by CTS (use the harmonic scapel)

Thoracoscopic surgery has been a common indication for mediastinal mature teratomas [1, 8]. It has been indicated when the size of benign teratomas is less than 6 cm in diameter [7–9]. In 2004, Akihiko Kitami et al. indicated larger size (but still less than 10 cm) [15]. However, many other factors such as the real lesions in the operation, the experience of the surgeon, available equipments and the supportive devices will also affect thorascopy decision. In our study, the largest tumor was removed by thoracoscopic surgery had the sizes of 8.0 cm × 9.0 cm on computerized tomography. In the operation, we conversed into mini-thoracotomy in 3 cases to remove tumor easier and avoid blood loss during the surgery (Fig. 2). In addition, the grades of adhesion of teratomas assessed during operation played an important role in the decision of conversion into mini-thoracotomy because of the risk of bleeding, which was also suggestion of Yang et al. (2005) [16]. Many studies suggested that conversion into mini-thoracotomy was needed if the tumor was more invasive in surrounding organs to ensurethe operation performed safely [9, 10, 16]. Teratomas composed mainly of germ cells in mediastinum, the detail pathology result is described in Table 2. and adhesion is the most common manifestation in these type of tumors. Chang et al. (2010) [18] and Moran et al. (1997) [19] had also reported that adhesion provided a large proportion of tumor characteristics. The results from our study showed that analysis on computerized tomography (tumor sizes, characteristics, and tumor boundary) and assessment of intraoperative tumor adhesion were two important factors to indicate thoracoscopic surgery approach in MMTs.

### Perioperative difficulties of this procedure

Selecting the patient was a very important step because the placement of trocars in operation depended on the location of the tumor in the mediastinum. In order to create the best working space for operations in mediastinal tumor surgery in general and MMTs in particular, trocars must be placed according to the “target triangle” principle [13] that based on “baseball diamond concept” of Landreneau et al. [14]. Our results corroborated this with all patients undergoing type IV approach of Sasaki [13] with complete tumor removals and no surgical complications. There were 3 cases with large tumors and severe adhesion needed to converse into mini-thoracotomy (5 - 8 cm). In addition, tumors located in the left of chest cavity usually had a longer duration of surgery than the right side because of the narrower operative space as a result of a heart occupancy.

Patient underwent general anesthesia with a double-lumen endotracheal tube. CO_2_ insufflation at 5 - 10 mmHg [11, 12] may help the operation performed easier. Adhesion was the one of most challenging in thoracoscopic surgery for MMTs because of the risk of bleeding and damage closed organs during operation [10, 16, 17]. Chang et al. advocated that in some patients, VATS was converted to an open thoracotomy because of strong adhesions and the difficulty in dissection of the involved vessels [17].

In addition, extraction of tumor contents (fluid) before continuing operation may have some advantages such as facilitating the dissection and removal of the tumor from the thoracic cavity, which is more convenient in operative manipulations and shortening operative time that resulted in application of thoracoscopic surgery for large mature cystic teratomas [8, 9]. The successful ratio in our study was 89.7% (complete removal of mediastinal mature teratomas by CTS and VATS). There were2 cases conversed from CTS into VATS because the operative manipulation could not be performed (01 tumor located in left side and had adhesion, 01 case had adhesion and perioperative bleeding).

We also compared the chest tube stay and the post-operative hospital stay between the two grops and saw a statistically significant difference (*p* <  0.05). Removing chest drain after VATS may benefit some patients by decreasing postoperative pain and shortening hospital stays, without increasing complications or compromising patient safety [20].

However, there is no difference in the operative time, although there was a statistically significant difference in tumor adhesion in CTS and VATS group (Table 1). We also found no statistically significant difference in the conversion to mini-thoracotomy rate between the two study groups (*p* = 0.078). However, we must emphasis again that this is a retrospective study and the number of patients is limited, so we need to follow up for better discussion.

## Conclusion

A thoracoscopic surgery for a mediastinal mature teratoma was a feasible choice. Challenging factors such as large tumors, intraoperative bleeding and strong tumor cell adhesion were considered handling by conversion to mini-thoracotomy that could ensure safe procedures and complete removal of tumors. Extraction of tumor contents might be performed for patients with large mature cystic teratomas to facilitate thoracoscopic surgery.

## Data Availability

Data sharing not applicable to this article as no data sets were generated or analyzed during the current study.
